# High fructose and streptozotocin induced diabetic impairments are mitigated by Indirubin-3-hydrazone via downregulation of PKR pathway in Wistar rats

**DOI:** 10.1038/s41598-021-92345-2

**Published:** 2021-06-21

**Authors:** Mary Priyanka Udumula, Sureshbabu Mangali, Jaspreet Kalra, Deepika Dasari, Srashti Goyal, Vandana Krishna, Srivarsha Reddy Bollareddy, Dharamrajan Sriram, Arti Dhar, Audesh Bhat

**Affiliations:** 1 Department of Pharmacy, Birla Institute of Technology and Sciences (BITS) Pilani, Hyderabad Campus, Jawahar Nagar, Shameerpet, Hyderabad, Telangana, Andhra Pradesh 500078 India; 2 Centre for Molecular Biology, Central University of Jammu, Jammu, UT of Jammu and Kashmir 184311 India

**Keywords:** Drug discovery, Type 2 diabetes

## Abstract

Metabolic disorders are becoming more common in young population due to increased consumption of carbohydrate rich diet, lack of physical activity and stress. Fructose is used as a sweetener in many carbonated beverages and is a known inducer of oxidative stress and hypertension. Up-regulation of the double-stranded RNA-dependent protein kinase (PKR) causes impairment in insulin signaling pathway and metabolic dysfunctions in type 2 diabetes mellitus. In the present study we investigated the role of PKR and associated pathways in high fructose (HF) and streptozotocin (STZ) induced diabetes and whether indirubin-3-hydrazone (IHZ), a novel PKR inhibitor can reverse the HF and STZ induced diabetic impairments in Wistar rats. Diabetes was induced by feeding rats 20% high fructose in drinking water for 6 weeks and by giving a single dose of STZ (35 mg/kg., i.p) at the end of week 5. Glucose and lipid levels were measured by using assay kits. Expression of PKR and its downstream genes were determined by immunohistochemistry, qRT-PCR and western blotting techniques. Histo-pathological studies were performed using H&E staining. Fibrosis was detected in insulin sensitive tissues and organs using Sirius red and Masson’s trichrome staining and apoptosis by TUNEL assay. HF and STZ induced hyperglycemia, fibrosis, oxidative stress, and inflammation in liver, pancreas, skeletal muscle and adipose tissue are mediated via PKR pathway and its downstream effectors, and these effects were attenuated by PKR inhibitor IHZ. Thus, inhibition of PKR can protect insulin sensitive organs and tissues from HF induced diabetic impairments via the inhibition of c-Jun N-terminal kinase (JNK) pathway.

## Introduction

Due to the specific metabolic fate and lipogenic property, fructose causes impairment in glucose metabolism, leading to insulin resistance, dyslipidemia and hepatic fibrosis, together with cardiac and renal dysfunctions^1–3^. During the course of excess sugar intake, wide range of stress and inflammatory responses are activated in metabolic tissues, such as pancreas, adipose tissue, skeletal muscle, and liver causing activation of c-Jun N-terminal kinase (JNK) and inhibitory κB kinase (IKK) pathways^4–6^. These pathways are important contributors in the progression and development of insulin resistance and type 2 diabetes (T2D) via activation of inflammatory cascade, impairment in insulin signaling, and disturbance in systemic glucose homeostasis and lipid profile. These processes further lead to increased reactive oxygen species (ROS) production and inflammatory response^7–9^.

Double-stranded RNA-dependent protein kinase (PKR), a well known regulator of inflammatory pathways is activated by interferons, toll like receptors, and nutrient excess. PKR is also an important signaling molecule for many downstream markers of inflammation, such as NF-ķB, JNK, IkB and apoptosis^10,11^. Nakamura et al. described the association of PKR in modulating the components of insulin signaling and attenuation of insulin action^12^. A significant activation of PKR was observed in liver and adipose tissue of obese mice overexpressing PKR, with an improvement in insulin signaling and amelioration of inflammatory responses in PKR deficient obese mice^12,13^. Although several mechanisms have been proposed, elucidating the role of PKR in T2D; however, the exact mechanism still remains to be understood, mainly due to high cost and limited range of selective PKR inhibitors currently available. Recently, our lab has reported a protective effect of indirubin derivative, indirubin-3-oxime (I3O) against PKR mediated deleterious effects in cardiomyocytes treated with high glucose^14^. Indirubin is the main active ingredient of Dangui Luhui Wan, a Chinese herbal drug and a new indirubin analogue, indirubin-3-hydrazone (IHZ) was recently characterized as a novel PKR inhibitor in cardiac myocytes by our group^15^. In our preliminary studies we found IHZ more potent inhibitor of PKR than I3O (unpublished data). However, the role of IHZ has not been explored in diabetes and its related complications. Therefore, we attempted to explore the PKR inhibitory property of IHZ in high fructose (HF) and streptozotocin (STZ) induced T2D animal model^16^ and are here for the first time reporting the therapeutic potential of IHZ in attenuating PKR mediated disruption of lipid and glucose homeostasis via downregulation of inflammatory cascade. Our results demonstrate the protective role of IHZ through restoration of functional alterations in pancreas, liver, skeletal muscle and adipose tissue in T2D rat model.

## Results

### IHZ attenuates metabolic impairments in HF and STZ induced diabetic rats

**Figure 1:**
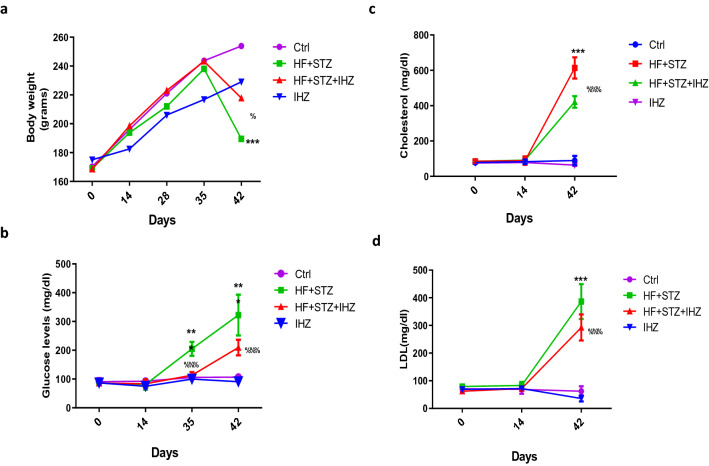
IHZ attenuates insulin resistance in HF with low STZ induced diabetic rats. Rats were fed with High Fructose (HF, 20%) water for 6 weeks and single dose of STZ (35 mg/kg, i.p.) was administered at the end of 5th week, with or without IHZ (25 mg/kg, p.o.) for 6 weeks. Analysis of body weight was done every week and biochemical parameters were carried out in plasma on 0, 14, 35 and 42 days from different animal groups. (**a**) Change in weight of animals during the study period. (**b**) Change in plasma cholesterol levels as measured using commercial kit over the course of the study on specified days. (**c**) Change in plasma glucose levels measured by glucometer on specified days. (**d**) Levels of low density Lipids (LDL) measured with commercial kit. Data is presented as mean ± SD of n = 6 animals for each group. ***P < 0.001 vs control group; %P < 0.05, %%%P < 0.001 vs HF + STZ group.

In this study, we investigated whether IHZ could attenuate hyperglycemia in HF and STZ induced diabetic rats. As a part of this investigation, body weight, blood glucose and lipid profile were considered as indicators of IHZ’s efficacy. Body weight, initially up to 35th day of the study showed no significant change in all the study groups, however, a significant decrease in body weight was observed in the HF + STZ group on the 42nd day when compared with the control group (p < 0.001) and a significant gain in the HF + STZ + IHZ group when compared with the HF + STZ group (p < 0.05) (Fig. 1a). Fasting glucose levels (Fig. 1b) increased significantly on 35th (p < 0.001) and 42nd day (p < 0.001) in the HF + STZ treated group when compared with the control group, and were attenuated significantly in the HF + STZ + IHZ treated group when compared with the HF group (p < 0.001). The levels of total cholesterol (Fig. 1c), low density lipoprotein (LDL) (Fig. 1d), aspartate aminotransaminase (AST) (a liver damage biomarker), and triglyceride (TG) (Suppl. Table 1) were found significantly higher in the HF + STZ group on day 42 when compared with the control group (p < 0.001) and were attenuated significantly in the HF + STZ + IHZ (Fig. 1c,d and Suppl. Table 1). High density lipoprotein (HDL) on the other hand showed a significant decrease in the HF + STZ group when compared with the control group (p < 0.001) and a significant increase in the HF + STZ + IHZ group when compared with the HF + STZ group (p < 0.01) (Suppl. Table 1). We next calculated the TG/HDL and TC/HDL ratios, as these ratios are used as a measurement of insulin resistance^17–19^. We found both ratios high in the HF + STZ group (TG/HDL = 17.6 and TC/HDL = 102.1) as compared to the control group (TG/HDL = 0.54 and TC/HDL = 3.7) and substantially low in the HF + STZ + IHZ than the HF + STZ group (Suppl. Table 2). Although we did not measure insulin levels in study animals; however, the ratios indicate an onset of insulin resistance in the HF + STZ group and a reversal in the HF + STZ + IHZ group.

### IHZ attenuates PKR expression, inflammation and fibrosis in the skeletal muscles of diabetic rats

**Figure 2:**
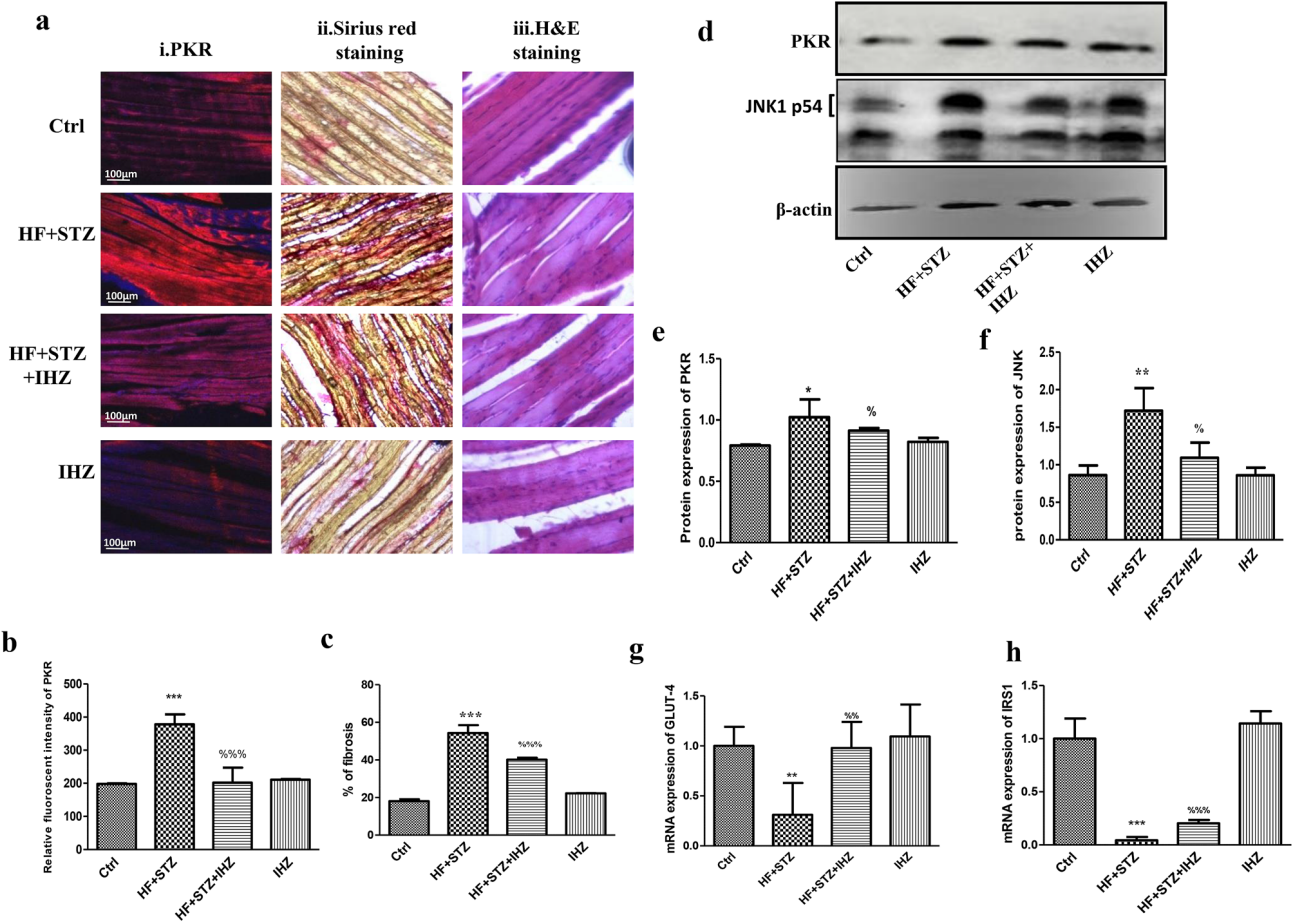
IHZ attenuates insulin resistance, inflammation & fibrosis via inhibition of JNK in skeletal muscle tissue of diabetic rats. (**a.i**,**b**) PKR expression levels in skeletal muscle as detected by immunohistochemistry. (**a.ii**,**c**) Picro-Sirius red staining was done to assess the extent of fibrosis and collagen deposition. (**a.iii**) H&E staining was done to observe changes in tissue morphology after treatment (**d**–**f**) Western blots showing the levels of PKR and JNK and their quantitative data. β-actin was used as loading control. (**g**) mRNA levels of *GLUT-4* in skeletal muscle as assessed by qRT-PCR. (**h**) mRNA levels of *IRS-1* in skeletal muscle as assessed by qRT-PCR. Data is presented as mean ± SD of n = 3 animals for each group. At the end of 6th week, rats were sacrificed and tissues were isolated. *P < 0.05, **P < 0.01, ***P < 0.001 vs control group; %P < 0.05, %%P < 0.01, %%%P < 0.001 vs HF + STZ group.

After testing the efficacy of IHZ, we checked if HF upregulates PKR expression in the skeletal muscle and whether IHZ could attenuate it. Post HF + STZ induced diabetes, we detected a significant increase in PKR levels in the skeletal muscles of diabetic rats when compared with the control group, using immunohistochemistry (IHC) (Fig. 2a, left panel and b) and western blotting techniques (Fig. 2d top panel and e). The levels were significant attenuated in the HF + STZ + IHZ group when compared with the HF + STZ group (Fig. 2a,b,d,e). The percentage of fibrosis in skeletal muscle was detected by Picro-Sirius red staining (Fig. 2a middle panel) and showed a significant increase in the HF + STZ treated group when compared with the control group, whereas the same was significantly reversed in the HF + STZ + IHZ treatment group (Fig. 2c). We also measured the expression of JNK in the different treatment groups and found a significant increase in the HF + STZ group when compared with the control group and a significant decrease in the HF + STZ + IHZ group when compared with the HF + STZ group (Fig. 2d,f). Following this, we detected the mRNA levels of insulin signaling genes *GLUT-4* and *IRS-1* and found a significant decrease in their levels in the HF + STZ group and normal levels after treatment with IHZ (Fig. 2g,h).

### IHZ attenuates apoptosis and fibrosis in the pancreatic tissue of diabetic rats

**Figure 3:**
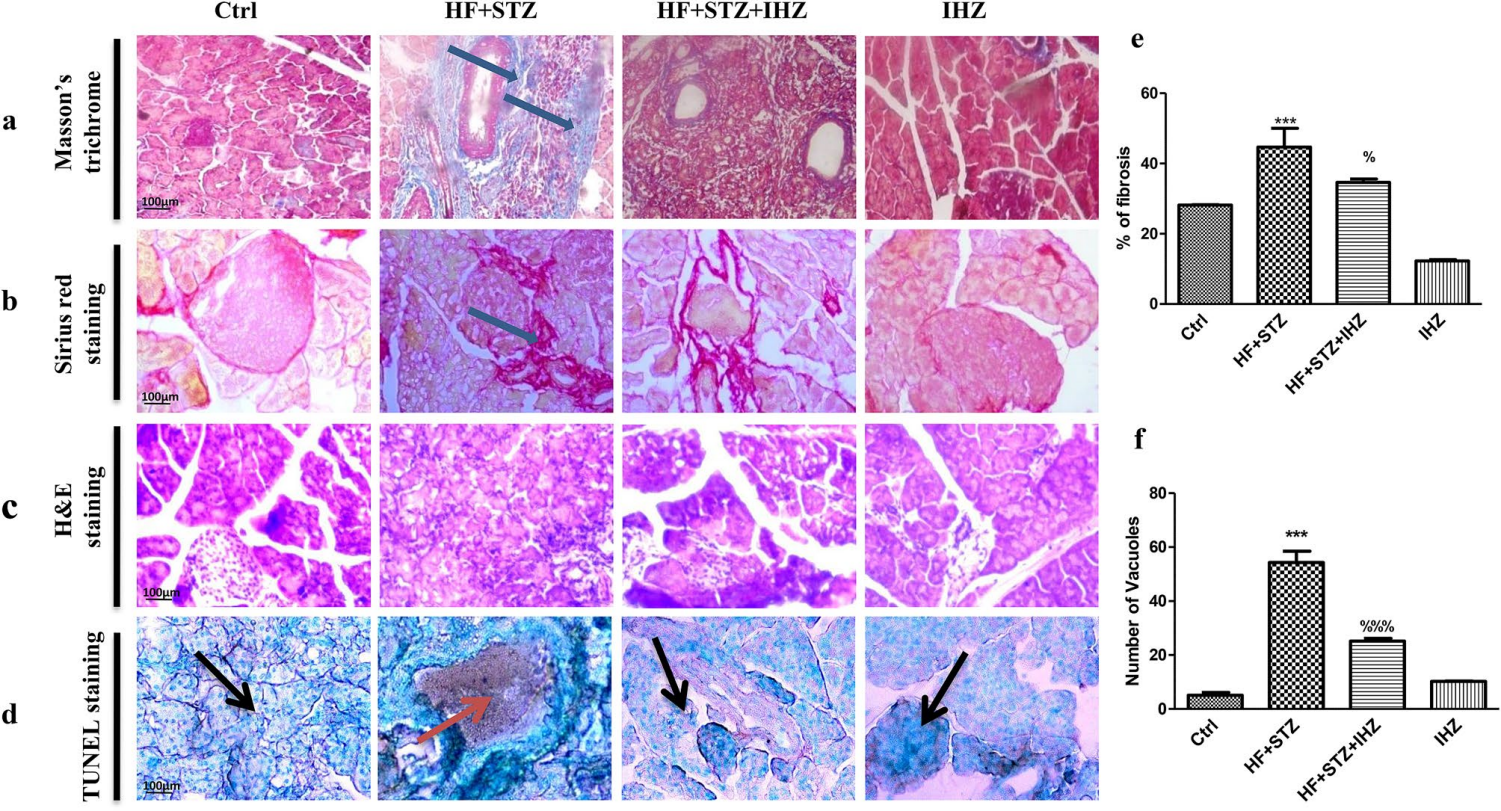
IHZ attenuates islet damage (apoptosis) and fibrosis in pancreatic tissue of diabetic rats. (**a**,**b**) Sections of pancreatic tissue stained with Masson’s trichrome and Picro-Sirius red staining, respectively to detect fibrosis (blue coloration, red coloration marked with arrows). (**c**) Sections of pancreatic tissue stained with H&E staining to detect cell morphology and number of vacuoles. (**d**) Representative images of TUNEL assay to assess apoptosis (apoptotic sites marked with arrows). (**e**,**f**) Quantitative data from figures (**a/b**,**c**). Data is presented as mean ± SD of n = 3 animals for each group. ***P < 0.001 vs control group, %P < 0.05, %%P < 0.01 vs HF + STZ group.

Figure 3a–d are representative images of staining to detect collagen deposition, cell morphology, fibrosis and apoptosis in the pancreatic tissue of different treatment groups. The percentage of fibrosis (Fig. 3a,b,e) and tissue degeneration (no of vacuoles) (Fig. 3c,f) in the pancreatic tissue of HF + STZ treated rats was found significantly higher when compared with the control group, whereas the percentage of fibrosis decreased significantly in the pancreatic tissue of the HF + STZ + IHZ treated group when compared with the HF + STZ group. We further investigated the effect of IHZ on apoptosis by using in-situ TUNEL assay kit. Apoptotic cells, specifically pancreatic islets were stained in brown color (indicative of apoptosis), which was found more prevalent in the HF + STZ treated group than in the HF + STZ + IHZ treated group (Fig. 3d).

### IHZ attenuates HF + STZ induced oxidative stress and JNK signaling pathway in pancreatic tissue

**Figure 4:**
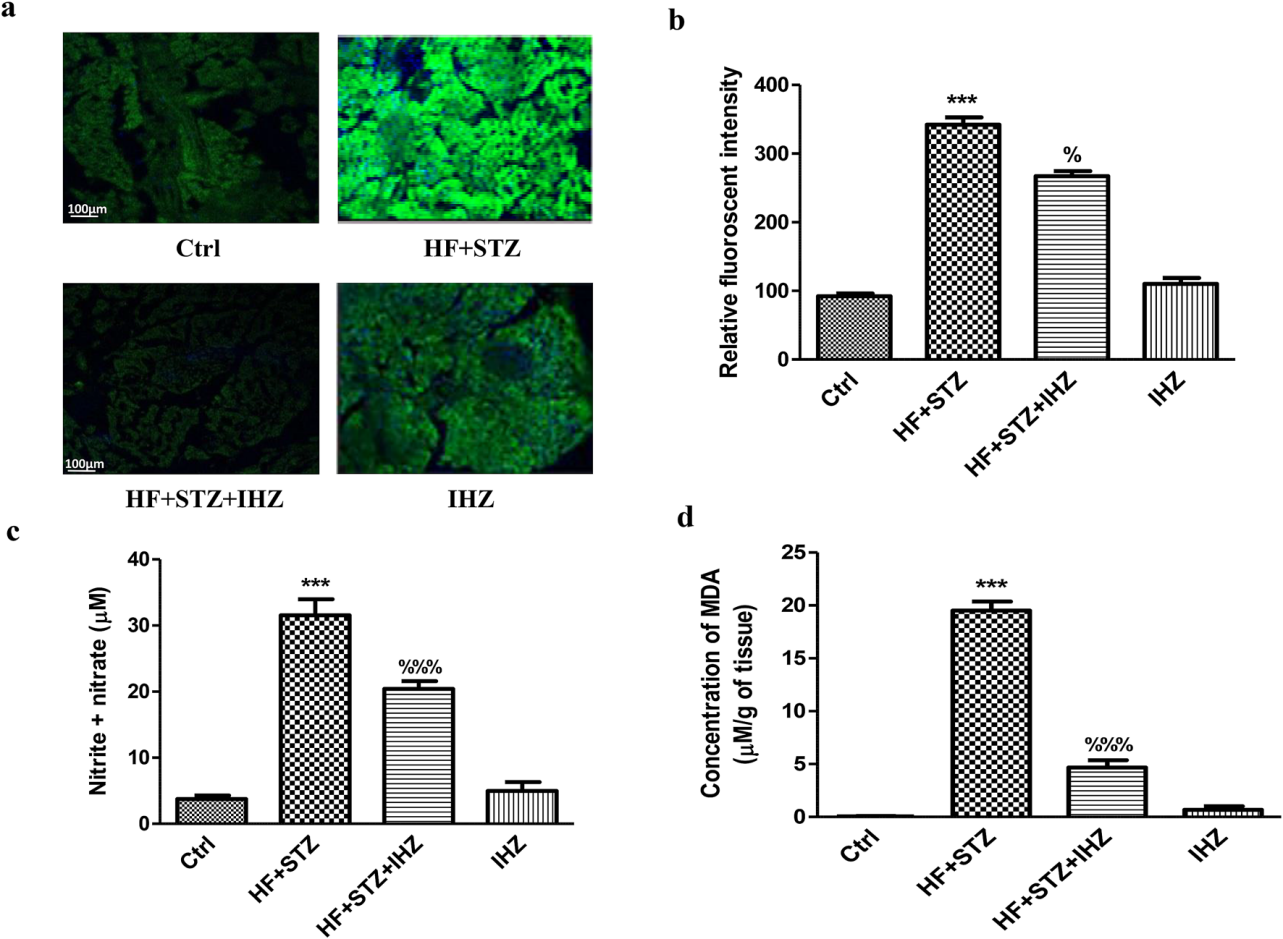
IHZ attenuates islet damage & fibrosis in pancreatic tissue of diabetic rats by inhibition of oxidative stress. (**a**) Representative images of histological pancreatic sections incubated with DCFDA to assess oxidative stress (high green fluorescence represents increased ROS generation). (**b**) Relative fluorescence intensity plotted from (**a**). (**c**) Nitrite or nitrate levels measured by Griess assay. (**d**) Lipid peroxidation levels measured by TBARS assay. Data is presented as mean ± SD of n = 3 animals for each group. ***P < 0.001 vs control group; %P < 0.05, %%%P < 0.001 vs HF + STZ group.

**Figure 5:**
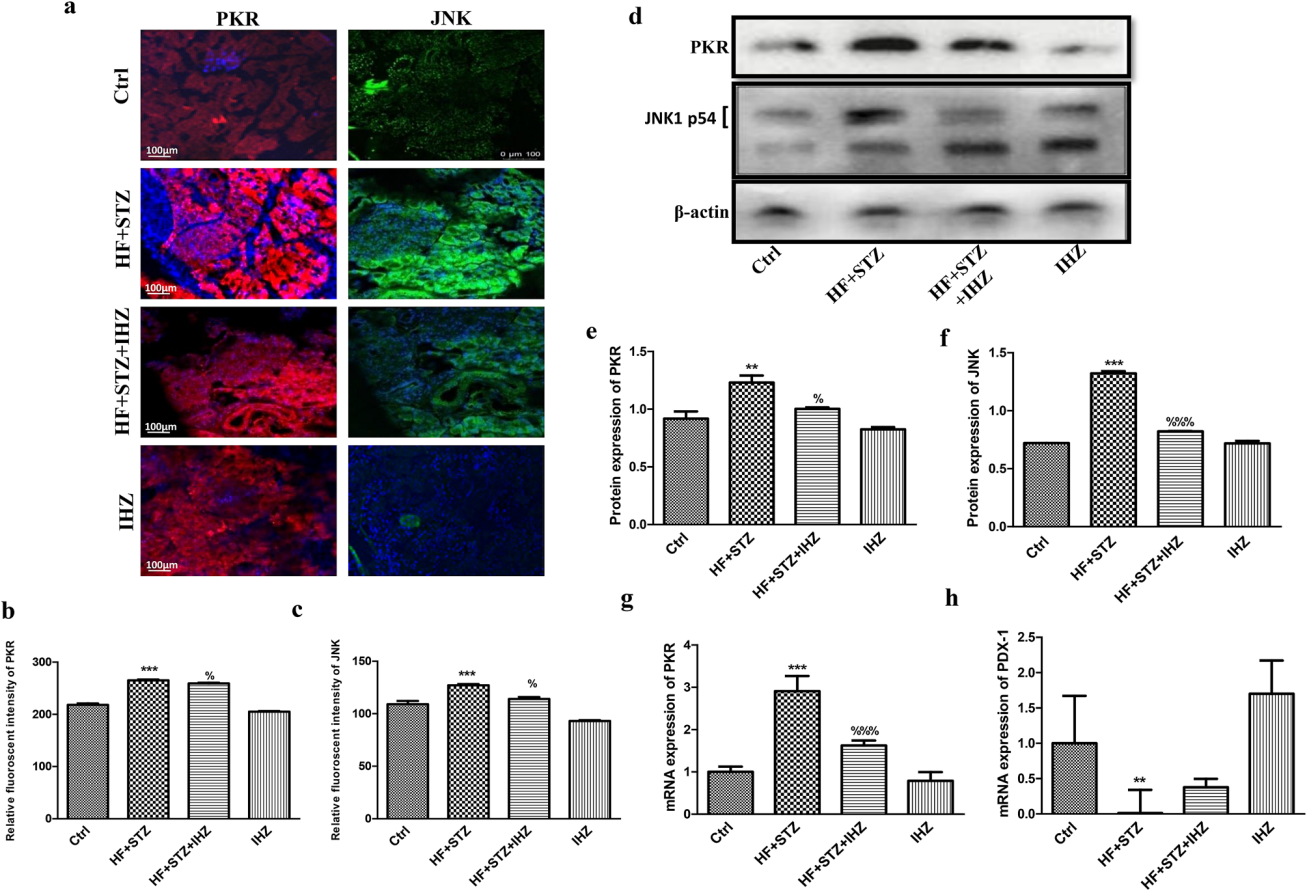
IHZ attenuates islet damage and fibrosis in pancreatic tissue of diabetic rats via inhibition of JNK signaling pathway. (**a**) PKR and JNK protein expression in pancreatic tissue detected by immunohistochemistry using suitable antibodies (representative images). (**b**,**c**) Quantitative data of (**a**). (**d**) Representative western blots showing the levels of PKR and JNK. β-actin was used as loading control. (**e**,**f**) Quantitative data of (**d**). (**g**) mRNA levels of *PKR* as detected by qRT-PCR. (**h**) mRNA levels of *PDX-1* detected by qRT-PCR (**d**); Data is presented as mean ± SD of n = 3 animals for each group. **P < 0.01, ***P < 0.001 vs control group; %P < 0.05, %%%P < 0.001 vs HF + STZ group.

HF is a known inducer of oxidative stress. Here we investigated whether IHZ can reverse the HF induced ROS production in the pancreatic tissue. To determine if HF induces oxidative stress, we performed the dichlorofluorescein diacetate (DCFDA) staining assay on pancreatic tissue sections (Fig. 4a) and measured the levels of nitrite & nitrate in the homogenized pancreatic tissue using the Griess reagent (Fig. 4c). As shown in Fig. 4a and b, ROS levels were found significantly increased in the HF + STZ group when compared with the control group (p < 0.001) and significantly reduced in the HF + STZ + IHZ group when compared with the HF + STZ group (p < 0.05). Increased levels of nitric oxide are considered as one of the oxidative stress markers. Like ROS, significantly higher levels of induced nitric oxide were found in the HF + STZ group when compared with the control group (p < 0.001) and significantly lower levels in the HF + STZ + IHZ group when compared with the HF + STZ group (p < 0.001) (Fig. 4c). A third marker of oxidative stress, the malondialdehyde (MDA), which is a byproduct of lipid peroxidation (LPO) was also measured and found significantly elevated in the HF + STZ group than in the control group (p < 0.001) and significantly attenuated in the HF + STZ + IHZ treatment group when compared with the HF + STZ group (p < 0.001) (Fig. 4d). To further delineate the underlying signaling mechanism of how PKR is involved in diabetes and the role of IHZ, we assessed the effect of IHZ on the expression of PKR and JNK in the pancreatic tissue both at protein as well as mRNA levels, as PKR is involved in multiple signaling cascades, including the JNK signaling pathway and the role of JNK in diabetes and inflammation is well documented^12^. As shown in Figs. 5a (left panel),b,d,e, and g, the expression of PKR, as assessed by immunohistochemistry (Fig. 5a,b), western blotting (Fig. 5d,e), and qRT-PCR (Fig. 5g) was found significantly higher in the HF + STZ group when compared with the control group (p < 0.01) and at comparable levels with the control group in the HF + STZ + IHZ group. These data suggest a transcriptional upregulation of *PKR* gene in the pancreas under diabetic condition. To get more insight in to the effect of PKR on JNK expression, we performed immunohistochemical (ICH) analysis of pancreatic sections using anti-JNK antibody. As shown in Fig. 5a (right panel) and c, the relative fluorescent signal was significantly higher in the HF + STZ group when compared with the control group (p < 0.001) and less intense in the HF + STZ + IHZ group when compared with the HF + STZ group (p < 0.05). Likewise, western blotting analysis also demonstrated a similar trend of JNK expression in the pancreatic tissue (Fig. 5d middle panel, f), thus validating our ICH results. It has previously been reported that pancreatic and duodenal homeobox 1 (PDX-1) positively regulates insulin gene expression and β-cell function. To check the role of PDX-1 in the HF + STZ diabetic model, we measured the mRNA expression levels of *PDX-1* in our samples. As shown in Fig. 5h, we detected a significant reduction in the mRNA levels of *PDX-1* in the HF + STZ group when compared with the control group (p < 0.01) and at comparable level in the HF + STZ + IHZ group. These data suggest that PKR is inducing diabetes by activating JNK signaling and by downregulating *PDX-1* gene expression in the pancreas.

### IHZ ameliorates hepatic fibrosis, morphological changes and adipose tissue alterations in diabetic rats through the inhibition of JNK signaling pathway

**Figure 6:**
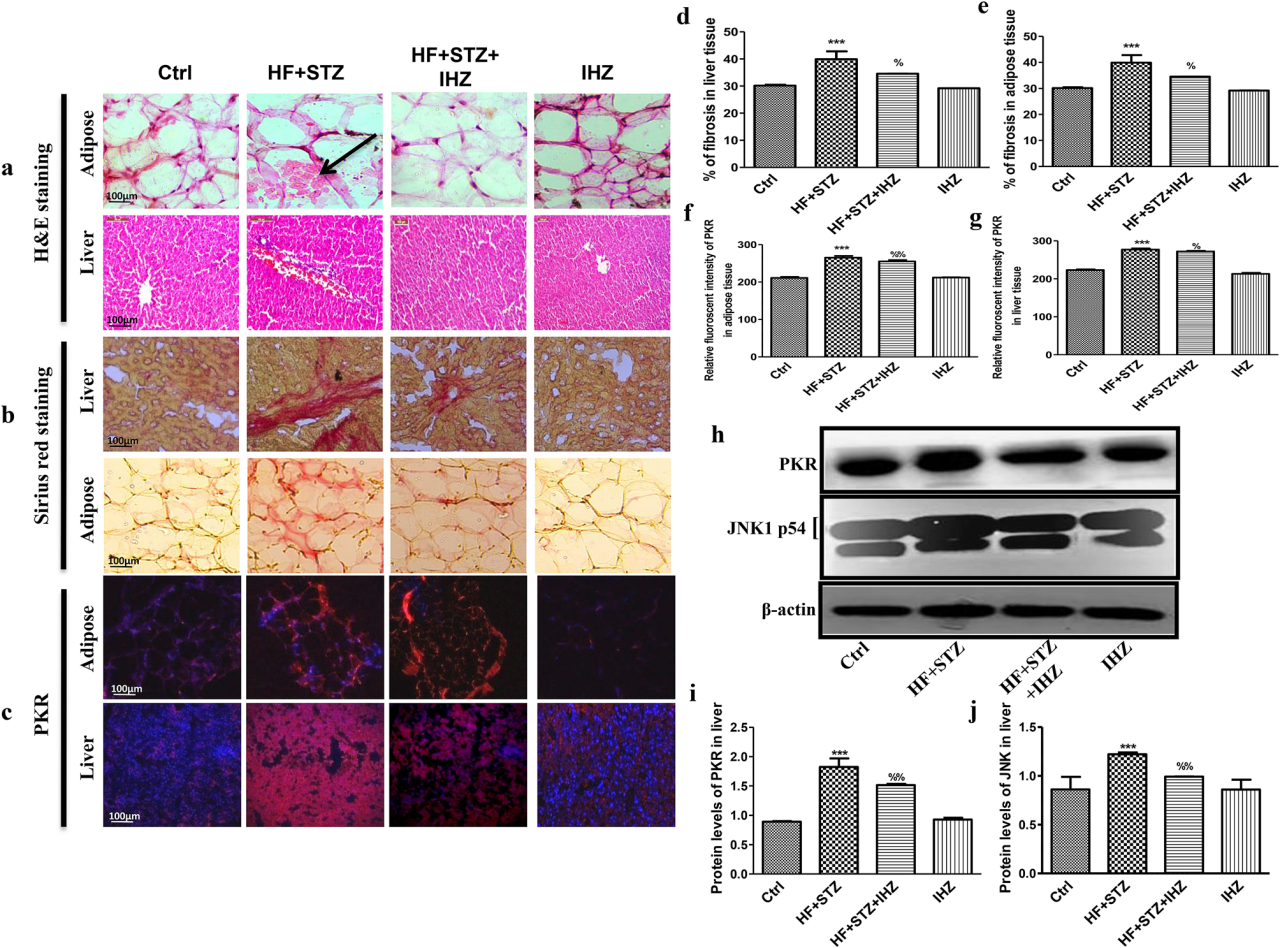
IHZ ameliorates hepatic fibrosis, morphological changes and adipose tissue alterations in diabetic rats by the inhibition of JNK signaling pathway. To detect morphological changes H&E staining was performed (**a**, black arrow shows change in morphology); Picro-Sirius red staining was performed to assess the extent of fibrosis and collagen deposition (Red color indicated depositions of collagen) (**b**); PKR expression in adipose and liver tissue was determined by immunohistochemistry using anti-PKR antibody (**c**); Graphs representing the quantitative data of the staining assays (**d**–**g**). Representative western blots showing protein levels of PKR and JNK in liver tissue of different treatment groups (**h**) and the quantitative analysis of these blots (**i,j**). Data is presented as mean ± SD of n = 3 animals for each group. ***P < 0.001 vs control group; %P < 0.05, %%P < 0.01 vs HF + STZ group.

To check whether diabetes also causes fibrosis and morphological changes in other insulin sensitive organs, such as adipose tissue and liver, we performed similar staining experiments on the tissue sections of these organs, as was done on pancreas and skeletal muscles. As shown in Fig. 6a, morphological changes were observed in the liver and adipose tissue of the diabetic rats, which were ameliorated after IHZ treatment. Likewise, the level of fibrosis in both liver as well as adipose tissue was significantly higher in HF + STZ group than in any other group, thus suggesting that IHZ effectively reverses diabetes induced tissue fibrosis (Fig. 6b,d,e). This is concurrent with the increased protein levels of PKR in the liver of the HF + STZ treated rats and decreased levels in the HF + STZ + IHZ treated rats (Fig. 6c,g,h,i). A similar trend was observed in the adipose tissue (Fig. 6c,f). Next we looked at the expression levels of JNK protein. As shown in Fig. 6h and j, the expression levels were found significantly higher in the liver tissue of HF + STZ treated rats than in the control group, whereas this expression was attenuated in the liver of the IHZ treated diabetic rats. These findings are in accordance with the previously published data, where high fructose intake has been associated with liver fibrosis^20,21^.

## Discussion

Obesity, insulin resistance, type 2 diabetes, and diabetes associated cardiovascular diseases are major global health problems with limited therapeutic options^18^. Previously, two PKR inhibitors; imoxin and 2-aminopurine have been shown to reduce inflammation, insulin resistance, and glucose intolerance in obese (ob/ob) mice^12^. Numbers of PKR inhibitors are currently being tested as PKR is involved in multiple pathological conditions and diseases, including obesity and diabetes^22^. PKR inhibitors, thus are emerging as novel drugs to treat T2D, metabolic disorders, and cancer; however, extensive research is needed to fully understand the underlying mechanism of action of these inhibitors. Apart from imoxin and 2-aminopurine, indirubin-3-oxime, a derivative of indirubin has been reported as novel PKR inhibitor^14^. Previously, our lab reported the effect of a newly synthesized derivative of indirubin, indirubin-3-hydrazone on high glucose induced apoptosis and oxidative stress^15^. However, the effect of PKR inhibitors on insulin resistance in skeletal muscle, liver and the relationship between PKR and pancreatic β-cell mass has not been reported yet. Therefore, in the present study we investigated whether inhibition of PKR pathway can improve HF and STZ induced metabolic alterations in skeletal muscle, liver and adipose tissue of diabetes rats. First, we investigated the effect of IHZ on biochemical parameters such as glucose, TC, TG, HDL, LDL and AST levels and noticed a significant improvement in all the parameters upon inhibition of PKR by IHZ in comparison to the diabetic group (Fig. 1). These findings are in agreement with our previous study, showing impairment in insulin signaling pathway via the activation of PKR in L6 skeletal muscle cells^23^. Since this is a first study on IHZ treated diabetic rats; therefore, we monitored body weight of animals in the different study groups. The body weight decreased drastically as expected in the HF + STZ group in comparison to the other treatment groups on day 42nd. Surprisingly, the loss of body weight in the IHZ only treated animals started early in the study (from day 14) in comparison to the other groups on their respective day of data collection (Fig. 1a). This is most probably due to reduction in adipose tissue mass caused by Indirubin treatment, as reported previously in mice^24^. The percentage of fibrosis also showed significant decrease in skeletal muscle of diabetic rats treated with IHZ. Next we checked the protein and mRNA levels of PKR in skeletal muscle, liver, pancreas and adipose tissue and found that PKR was significantly upregulated in the HF + STZ rats, whereas this upregulation was significantly attenuated in the HF + STZ + IHZ rats, indicating that IHZ is effectively blocking HF + STZ induced PKR upregulation (Fig. 2). Interestingly, we also found increased levels of *GLUT-4* and *IRS-1* mRNA upon PKR inhibition in the skeletal muscle of diabetic rats (Fig. 2g,h), which correlates well with the protective effect of IHZ against hyperglycemic conditions. This further supports the therapeutic potential of IHZ in maintaining the glucose homeostasis and insulin sensitivity in HF induced hyperglycemia. Elevated levels of lipid profile markers and hepatic insulin resistance noticed in T2D are possibly due to the increased deposition of triglycerides in liver and production of free fatty acid metabolites. These processes further induce the production of ROS and elicit inflammatory response in metabolic tissues^7–9^ and insulin resistance^23^. To test this in our model, we checked the levels of fibrosis in liver, pancreas, and adipose tissue and apoptosis in pancreatic tissue of HF + STZ group and compared them with the HF + STZ + IHZ treated group. As is evident from our data (Fig. 3), levels of fibrosis significantly increased in the HF + STZ rats and IHZ effectively reversed this diabetes induced fibrosis. Furthermore, we looked at the levels of ROS, nitrate, nitrite, and LPO in the pancreatic tissue and observed a significant increase in the diabetic rats and a significant reduction in the IHZ treated diabetic rats (Fig. 4). This suggests that IHZ is protecting pancreatic cells from diabetes induced damage and fibrosis by suppressing oxidative stress. PDX-1 is necessary for pancreatic development and β-cell function and it has been reported that mutations and silencing of *PDX-1* in β-cells induces diabetes^25^. In our study, we detected significantly lower levels of *PDX-1* mRNA in the pancreatic tissue of diabetic rats and approximately 50% recovery in IHZ treated diabetic rats (Fig. 5h). Surprisingly we also found slight upregulation of PDX-1 in the IHZ only treatment group. Based on these observations it is possible that prolonged inhibition of PKR may lead to the upregulation in PDX-1 expression, which needs to be investigated in future. PKR regulates the various inflammatory pathways. The JNK pathway is one the downstream inflammatory pathway and its silencing is responsible for decreased adiposity, improved insulin sensitivity and insulin signaling. JNK is a negative regulator of insulin action by inducing stress and inflammation^26^. As expected, the expression of JNK was found upregulated in skeletal muscles, pancreas, and liver of diabetic rats and the same was attenuated by IHZ (Fig. 5). This further supports the idea that inhibition of PKR by IHZ could alleviate diabetes induced, metabolic impairments and inflammation and fibrosis in the insulin sensitive organs and tissues, such as skeletal muscles, adipose tissue, pancreas, and liver of the diabetic patients.

## Conclusion

Based on these observations that diabetes induces impairment in skeletal muscles, hepatic tissue and other insulin sensitive organs and tissues via the activation of PKR and that inhibition of PKR by IHZ protects these tissues and organs from diabetes induced fibrosis, inflammation, oxidative stress and JNK activation, it is safe to conclude that inhibition of PKR with selective inhibitors could confer protection against the diabetes induced impairments of the insulin sensitive tissues and organs via the inhibition of JNK pathway. However, further validation is needed in order to develop better and safer PKR inhibitor(s).

## Materials and methods

### Ethical statement

The animal experiments were done in accordance with the Committee for the Purpose of Control and Supervision of Experiments on Animals (CPCSEA) guidelines. All protocols for the experiments were approved by the institutional animal ethics committee (IAEC) of BITS-Pilani, Hyderabad Campus (letter number HYD/IAEC/2016/06).

### Chemicals and kits

Commercially available kits for assessment of glucose, low density lipoproteins (LDL), high density lipoproteins (HDL), triglycerides (TG’s), total cholesterol (TC), and aspartate transaminase (AST) were purchased from Tulip diagnostics (P) Ltd. (Mumbai, India) and Arkray Healthcare Pvt. Ltd, (Surat, India). Streptozotocin (STZ), hematoxylin and eosin (H&E), Sirius red, poly-l-lysine coating solution, Griess reagent and primers were purchased from Sigma Aldrich (St. Louis, Missouri, United States). Anti- PKR, JNK, and β-actin antibodies and the secondary antibodies were purchased from Santa Cruz Biotechnology (CA, USA). Masson’s trichrome and TUNEL assay kits were purchased from Abcam (Milton, Cambridge, UK). iScript cDNA synthesis kit, iTaq Universal SYBR® Green Supermix and the Immun-Blot® PVDF Membrane were purchased from Bio-Rad (Hercules, CA, USA). Trizol reagent was purchased from In-vitrogen.

### Animal model

All the animal experiments were conducted in the animal house of BITS Pilani Hyderabad campus with the protocol number HYD/IAEC/2016/06. Wistar male rats of 5–6 weeks old with a body weight of 180–220 gms were randomly divided into 4 groups as (n = 6 in each group): control group (C), diabetic group (HF + STZ), diabetic + indirubin-3-hydrazone group (HF + STZ + IHZ) group, indirubin-3-hydrazone (IHZ) alone group. Animals were housed in a temperature and humidity controlled room (22 ± 1 °C, 45–60% humidity) with a set of 12 h light–dark cycle. The rats were fed with standard rat pellet diet ad libitum. Animals were euthanized at the end of the study by exposing them to carbon dioxide.

### Induction of diabetes

To develop diabetic animal model, hyperglycemia was induced by feeding rats with 20% high fructose water, followed by induction of late onset of type 2 diabetes by administering low dose of STZ as described previously^15,16^. Study animals were divided into four groups. Group I (control) rats were fed with normal drinking water ad libitum, followed by the administration of citrate buffer after five weeks. Group II (diabetic) and Group III (diabetic + IHZ) rats were fed with 20% high fructose in drinking water throughout the study schedule (6 weeks). In the treatment schedule, at the end of fifth week, a single low dose of STZ (35 mg/kg., i.p) was administered through i.p route to Group II and Group III anaimals. STZ was dissolved in 0.1 M citrate buffer (pH4.4). Group III animals received IHZ at a dose of 2.5 mg/kg., P.O from day one of treatment schedule. Group IV (IHZ) rats received IHZ only at the above mentioned dose. IHZ dose was chosen based on the literature and our previous study^15,24^.

### Biochemical estimations and assessment of lipid profile

Fasting glucose levels (plasma), aspartate transaminase (AST) activity, total cholesterol (TC), Triglycerides (TGs), high density lipoprotein (HDL) and Low density lipoprotein (LDL) were assessed by using commercially available kits and the assays were performed according to the manufacturer’s protocol (Tulip diagnostics (P) Ltd, Mumbai, India and Arkray Health care Pvt. Ltd, Surat, India). Body weight was measured on 0, 14, 35 and 42nd day. Blood glucose levels were measured on day 0, 14, 35 and 42nd day. Cholesterol and LDL level were measured on 0, 14 and 42nd day respectively. Blood samples were collected by retro orbital method using capillary tubes and plasma was collected after adding 0.5 M EDTA.

### Estimation of MDA, nitrite and nitrate levels: markers for oxidative stress

The MDA, a by-product of lipid peroxidation was detected by thiobarbituric acid reactive species assay (TBARS) as described by Ohkawa et al.^27^. The concentration of TBARS was expressed as µmol of MDA per gram of tissue. Nitrite and nitrate levels were measured in the supernatant of tissue homogenate and absorbance was recorded at 540 nm using Spectramax plus 384 microplate reader (Molecular Devices, California, USA).

### Protein and gene expression studies

#### Western blotting

Western blotting was performed as described previously^28^. In brief, protein samples (40–50 µg) were collected from homogenized pancreas, skeletal muscles and liver. Tissue homogenates were prepared in tissue homogenizer containing protein lysis buffer (50 mM Tris–HCl, pH 7.5, 250 mM NaCl, 5 mM EDTA,50 mM NaF, and 0.5% Nonidet P-40; containing a protease inhibitor cocktail (Sigma Aldrich, India). Protein was separated on 8–10% SDS PAGE and transferred onto PVDF membrane. Membranes were blocked with 5% non fatty milk and incubated with primary antibody (1:1000) at 4 °C overnight followed by incubation with secondary antibody (1:3000) for 2 h. Later protein was detected on the membrane by enhanced chemiluminescence detector. All antibodies were procured from Santa Cruz Biotechnology, Santa Cruz, California, USA (anti-JNK [sc-137018], anti-PKR [sc-708], anti-actin [sc-47778], goat anti-mouse IgG-HRP [sc-2005]).

#### Real time quantitative PCR (qRT-PCR)

RNA extraction and qRT-PCR was performed as described previously^15^. The qRT-PCR of 42 cycles was done in the iCycler iQ apparatus (Bio-rad) in triplicates. Sequence of primers used in qRT‐PCR are as: *PDX1* Forward 5′-TGGAGCTGGCAGTGATGTTGA-3′ and Reverse 5′-TCAGAGGCAGATCTGGCCAT-3′; *PKR* Forward 5′-GCAGCAGTGGTTGGAAAAGA-3′ and Reverse 5′-TGTTGCAAGGCCAAAGTCTC-3′; *JNK* Forward 5′-TGGATTTGGAGGAGCGAACT-3′ and Reverse 5′-ACTGCTGTCTGTATCCGAGG-3′; Glut 4 Forward 5′-CGGGACGTGGAGCTGGCCGAGGAG-3′ and Reverse 5′-CCCCCTCAGCAGCGAGTGA-3′; IRS-1 5′-GCCAATCTTCATCCAGTTGC-3′ and Reverse 5′-CATCGTGAAGAAGGCATAGG-3′; *β-actin* 5′-GAGGCCCCTCTGAACCCTAA-3′ and Reverse 5′-ACCAGAGGCATACAGGGACAA-3′.

### Histopathological studies

Histopathological studies were performed on skeletal muscle, pancreas, adipose and liver sections to determine the changes in cellular morphology (H&E staining) and for the assessment of collagen deposition and fibrosis (Sirius red and Masson’s trichrome staining). The assays were performed as described previously^28^. Briefly, fixed and paraffinized tissues prepared from different organs of the rats were cut into 3-5 µm blocks using microtome. The tissue sections were then deparaffinised, rehydrated and stained with H&E stain (to detect changes in cellular morphology) and Picro-Sirius red (to detect collagen deposition) and Masson’s trichrome stains (to detect collagen deposition and tissue fibrosis). The stained sections were mounted on glass slides using DPX mounting solution. The sections were then examined under the microscope at 10X and 20X magnifications (Carl Zeiss Microscopy, Cambridge, USA). Results were quantified by ImageJ, image analysis software of NIH. Quantitative analysis of Sirius red and TUNEL stained images sections was performed by using ImageJ program. Fibrotic and apoptotic areas were measured across all treatment groups for comparison.

### Immunohistochemistry

Pancreatic, liver, skeletal muscle and adipose tissue sections (5 μm) were mounted on the coated slides. Tissue sections were deparaffinised and rehydrated, followed by antigen retrieval and incubation with 3 percent bovine serum albumin for 1 h at room temperature to block non-specific binding. IHC was performed to detect PKR and JNK expressions using anti-PKR and anti-JNK primary antibodies. A minimum of 3–6 slides per group and 6–10 fields per slide were evaluated under the high-power field (40X). Slides were examined using confocal microscope (Leica DMi8 confocal microscope, Germany) and quantified by ImageJ^23^.

### Statistical analysis

The collected data from multiple individual experiments were analysed using Graphpad Prism 6 (https://www.graphpad.com/scientific-software/prism/) and presented as mean ± standard deviation. ONE WAY ANOVA with post- hoc Bonferroni's test and Sidak’s multi comparison test was performed for statistical analysis. *p* value < 0.05 was considered as statistically significant. For quantification analysis, ImageJ software tool was used (https://imagej.nih.gov/ij/download.html).

### Animal studies

Animal studies were performed in compliance with the ARRIVE guidelines.

## Supplementary Information

Supplementary Information.
