# Publisher Correction: High fructose and streptozotocin induced diabetic impairments are mitigated by Indirubin‑3‑hydrazone via downregulation of PKR pathway in Wistar rats

**DOI:** 10.1038/s41598-021-96409-1

**Published:** 2021-08-19

**Authors:** Mary Priyanka Udumula, Sureshbabu Mangali, Jaspreet Kalra, Deepika Dasari, Srashti Goyal, Vandana Krishna, Srivarsha Reddy Bollareddy, Dharamrajan Sriram, Arti Dhar, Audesh Bhat

**Affiliations:** 1 Department of Pharmacy, Birla Institute of Technology and Sciences (BITS) Pilani, Hyderabad Campus, Jawahar Nagar, Shameerpet, Hyderabad, Telangana, Andhra Pradesh 500078 India; 2 Centre for Molecular Biology, Central University of Jammu, Jammu, UT of Jammu and Kashmir 184311 India

Correction to: *Scientific Reports* 10.1038/s41598-021-92345-2, published online 21 June 2021

The original PDF version of this Article contained an error in the Author Information section where Jaspreet Kalra was incorrectly indicated as an equally contributing author.

This error has now been corrected in the PDF version of the Article; the HTML version was correct from the time of publication.

