# Supplementary material for: High fructose and streptozotocin induced diabetic impairments are mitigated by Indirubin-3-hydrazone via downregulation of PKR pathway in Wistar rats
Source: Sci Rep. 2021 Jun 21;11:12924. doi: 10.1038/s41598-021-92345-2 (PMC8217483; doi:10.1038/s41598-021-92345-2)

**High fructose and streptozotocin induced diabetic impairments are mitigated by Indirubin-3-hydrazone via downregulation of PKR pathway in Wistar rats**

Mary Priyanka Udumula^1^**^#^**, Sureshbabu Mangali ^1^**^#^**, Jaspreet Kalra^1^**^#^**, Deepika Dasari^1^, Srashti Goyal^1^, Vandana Krishna^1^, Srivarsha Reddy Bollareddy^1^, Dharamrajan Sriram^1^, Arti Dhar^1*^, Audesh Bhat^2*^

^1^Department of Pharmacy, Birla Institute of Technology and Sciences (BITS) Pilani, Hyderabad Campus, Jawahar Nagar, Shameerpet, Hyderabad, Telangana-500078, India.

^2^Centre for Molecular Biology, Central University of Jammu, UT Jammu and Kashmir-184311, India

*Authors for correspondence:

| **Dr. Arti Dhar**  Department of Pharmacy, Birla Institute of Technology and Sciences Pilani, Hyderabad, Jawahar Nagar, Shameerpet, Hyderabad,  Andhra Pradesh 500078, India  Tel. 04066303647 / +919505563087  E-mail: [artidhar@hyderabad.bits-pilani.ac.in](mailto:artidhar@hyderabad.bits-pilani.ac.in) |
| --- |

**Dr. Audesh Bhat**

Centre for Molecular Biology

Central University of Jammu

UT of Jammu and Kashmir, India-184311

+91-9419532912

E-mail: [abhat@cujammu.ac.in](mailto:abhat@cujammu.ac.in)

**Suppl. Table 1: AST, HDL and Triglyceride levels in blood serum of rats.**

| **Biochemical parameter** | **Control** | **HF+STZ** | **HF+STZ+IHZ** | **IHZ** |
| --- | --- | --- | --- | --- |
| **AST** | 10.59 units/L ±0.0821 | 41.15 units/L ±1.11**^***^** | 30.21 units/L ±0.9**^%%%^** | 13.2 units/L ±0.118 |
| **HDL** | 24 mg/dL ±1.8 | 6 mg/dL ±2.7**^***^** | 11 mg/dL ±1.7**^%%%^** | 23 mg/dL ±2.91 |
| **Triglycerides** | 13.55 mg/dL ±2.9 | 105.78 mg/dL ±16.59*** | 90.06 mg/dL ±10.03 | 18.32 mg/dL ±4.2 |

Data presented here is of day 42 of the study. ***P< 0.001 versus respective control, %%%P<0.001 versus respective HF+STZ Data is expressed as mean ± SD of n=6 animals for each group. AST = Aspartate Transaminase, HDL = high density lipoproteins, HF = High Fructose, STZ = Streptozotocin, IHZ = Indirubin Hydrazone.

**Suppl. Table 2: TG/HDL and TC/HDL RATIOS**

| **Biochemical parameter** | **Control** | **HF+STZ** | **HF+STZ+IHZ** | **IHZ** |
| --- | --- | --- | --- | --- |
| TG/HDL | 0.54 | 17.6 | 8.18 | 1.5 |
| TC/HDL | 3.7 | 102.1 | 38.2 | 2.73 |

HDL = High-density lipoproteins, TC = total cholesterol, TG = triglyceride

**Suppl. Fig. 1:** Full length western blots of PKR, JNK, and β-actin protein expression of the main **figure 2d**. Please note that full length membranes with edges could not be provided as the membrane was cut before incubation with the selected antibodies.


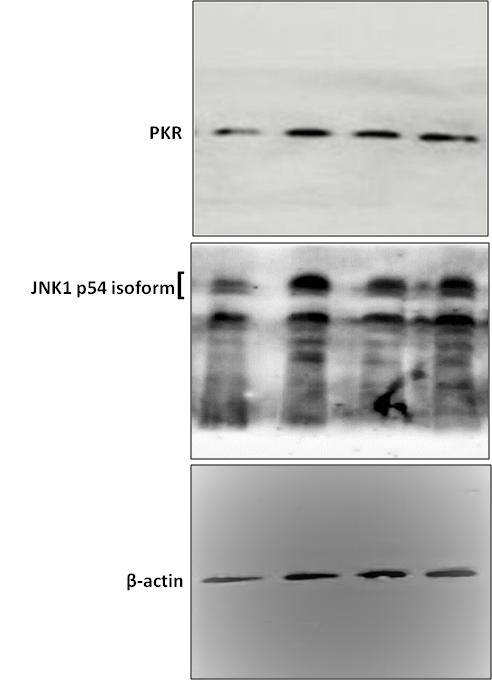


**Suppl. Fig. 2:** Full length western blots of PKR, JNK, and β-actin protein expression of the main **figure 5d**. Please note that full length membranes with edges could not be provided as the membrane was cut before incubation with the selected antibodies.


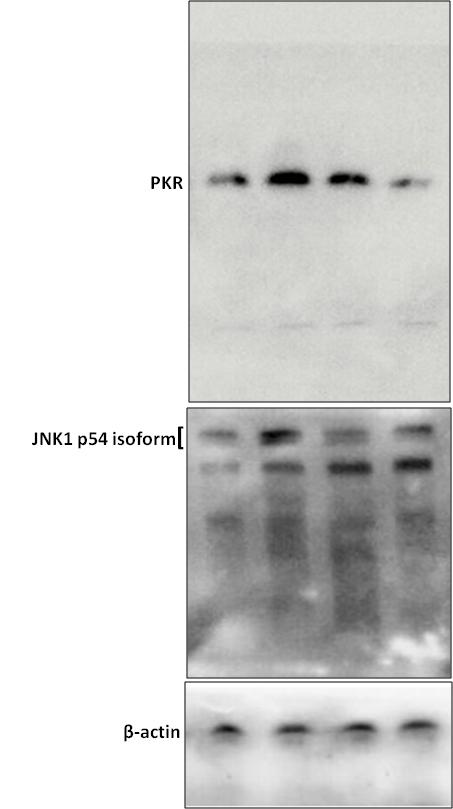


**Suppl. Fig. 3:** Full length western blots of PKR**,** JNK, and actin protein expression shown in the main **figure 6h**. Please note that full length membranes with edges could not be provided as the membrane was cut before incubation with the selected antibodies.

.


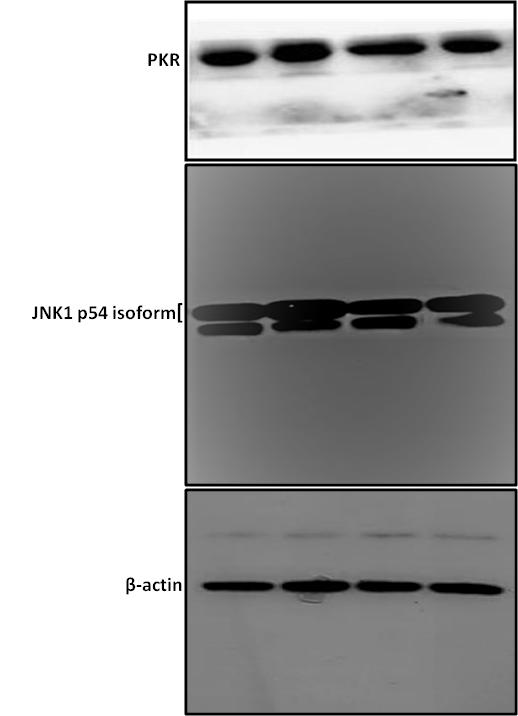

Supplement: Supplementary file 1 — Supplementary Information. [file 41598_2021_92345_MOESM1_ESM.docx]
